# Lysozyme-Induced Nephropathy: A Diagnosis Not to Forget

**DOI:** 10.7759/cureus.34344

**Published:** 2023-01-29

**Authors:** Núria Paulo, Pedro Baptista, Fernando Nogueira, Catarina Pereira, Ana Cerqueira, Ana Rocha

**Affiliations:** 1 Nephrology, Centro Hospitalar e Universitário de São João, Porto, PRT; 2 Hematology, Centro Hospitalar e Universitário de São João, Porto, PRT; 3 Internal Medicine, Centro Hospitalar e Universitário de São João, Porto, PRT; 4 Medicine, INEB-I3S, Porto, PRT

**Keywords:** case report, hematologic malignancies, lysozyme-induced nephropathy, acute kidney injury, lysozyme

## Abstract

Kidney injury in hematologic malignancies can manifest in many ways. We present a case report of a 44-year-old female with de novo acute myeloid leukemia (AML) and acute kidney injury. Following the etiological investigation, lysozyme-induced nephropathy was believed to be the most probable cause of renal injury. Intensive cytoreduction and chemotherapy were started and the patient's cytopenias and kidney injury have improved.

This case highlights the importance of recognizing lysozyme-induced nephropathy as a form of kidney injury in AML. Despite being frequently underrecognized, a precocious diagnosis may impact the patient’s prognosis.

## Introduction

Hematologic malignancies can affect all compartments of the nephron [1]. One important and usually underrecognized cause of kidney impairment in acute leukemias is lysozyme-induced nephropathy which involves tubular dysfunction [2]. Early recognition of this entity may impact a patient’s prognosis.

We present a case of a 44-year-old woman with de novo acute myeloid leukemia (AML), in whom lysozyme-induced nephropathy is believed to be the most probable cause of kidney injury.

This case report was previously presented as a meeting abstract at the 2022 Encontro Renalon November 17, 2022.

## Case presentation

A 44-year-old woman presented to the emergency room with one-month symptoms of fatigue, night sweats, and anorexia. She had a medical history of obesity and hypertension and she was being treated with lisinopril/hydrochlorothiazide 20/12.5 mg and alprazolam 0.5 mg. Physical examination revealed hemodynamic stability, palpable cervical adenopathy, and hepatosplenomegaly. Laboratory investigation showed hemoglobin 5.5 g/dL; leucocytes 340 x 10^9^/L (with 93% immature-appearing monocytoid cells), platelets 47 x 10^9^/L; lactate dehydrogenase 782 U/L. A bone marrow aspirate was performed and revealed a hypercellular marrow with replacement of the normal hematopoietic tissue by a population of neoplastic monoblasts (20.4%), promonocytes (4.8%), and, predominantly, monocytes (68.0%). Cytogenetic analysis showed a normal karyotype but molecular studies identified mutated nucleophosmin 1 (*NPM1*) in the absence of FMS-like tyrosine kinase 3 (*FLT3*) internal tandem duplication (*ITD*). Importantly, serum creatinine was 2.0 mg/dL; urea 51 mg/dL (previously available results in 2018 revealed normal kidney function); sodium 133 mEq/L, potassium 1.8 mEq/L, chlorine 92 mEq/L; phosphate 1.7 mg/dL, magnesium 1.2 mEq/L, ionized calcium 1.9 mEq/L and uric acid 12.6 mg/dL (Table 1). The patient was admitted to the hematology department with the diagnosis of AML with mutated *NPM1* and monocytic differentiation (acute monocytic leukemia, M5b morphology).

**Table 1:** Laboratory results at hospital admission. RBC – red blood cells; WBC – white blood cells

Parameter	Value (reference range)
Hemoglobin (g/dL)	5.5 (12.0-16.0)
Leucocytes (x 10^9^/L)	349.0 (4.0-11.0)
Differential blood count	
Neutrophils (%)	1.5 (53.8-69.8)
Monocytoid cells (%)	93
Lymphocytes (%)	4.5 (22.6-36.6)
Eosinophils (%)	0.5 (0.6-4.6)
Platelets (x 10^9^/L)	47 (150-400)
C-Reactive Protein (mg/L)	159.5 (<3.0)
Serum creatinine (mg/dL)	2.0 (0.5-0.9)
Serum urea (mg/dL)	51 (10-50)
Serum sodium (mEq/L)	133 (135-147)
Serum potassium (mEq/L)	1.8 (3.5-5.1)
Serum chlorine (mEq/L)	92 (101-109)
Serum phosphorus (mg/dL)	1.7 (2.7-4.5)
Serum magnesium (mEq/L)	1.2 (1.6-2.1)
Serum uric acid (mg/dL)	12.6 (2.3-6.1)
Lactate dehydrogenase (U/L)	729 (135-225)
Urine dipstick protein (g/L)	1+
Urine RBC (/uL)	22.9 (<20.0)
Urine WBC (/uL)	28.9 (<20.0)
24-hour Urine Protein (g/24h)	7.6
24-hour Urine Albumin (g/24h)	1.2

The urinary sediment contained no dysmorphic erythrocytes or erythrocyte casts and urinalysis showed 1+ protein by dipstick. A kidney ultrasound revealed normal-sized kidneys with normal parenchymal differentiation and ruled out hydronephrosis. During the inpatient stay, polyuria (urine output was 6 to 8 liters per day) and sustained electrolyte disturbances (hypokalemia, hypophosphatemia, and hypomagnesemia) requiring high-dose supplementation were identified. Total proteinuria was 7.6 g/24 hours and total albuminuria was 1.5 g/24 hours. These findings raised the hypothesis of tubular dysfunction and, therefore, serum and urine lysozyme levels were measured: serum lysozyme was 40 mg/L (range 4-13 mg/L) and urine lysozyme was > 105.0 mg/L (range < 3.6 mg/day). A kidney biopsy was not performed considering the patient’s frailty status at that moment, the hemorrhagic risk, and the fact that the results would not change the treatment strategy at that time. Nevertheless, lysozyme-associated renal dysfunction was assumed to be the most probable cause of kidney injury.

**Figure 1:**
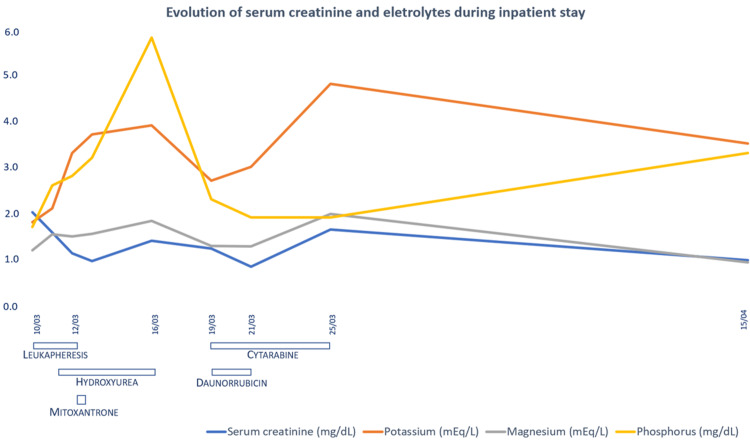
Evolution of serum creatinine and electrolytes during inpatient stay in relation to treatment.

Due to the extreme hyperleukocytosis, urgent cytoreduction was started with leukapheresis, mitoxantrone, and hydroxyurea. A few days after admission intensive induction chemotherapy with cytarabine and daunorubicin was started. Subsequently, kidney function normalized with progressive improvement of electrolyte disturbances. At discharge, laboratory results showed leucocytes 4.1 x 10^9^/L, serum creatinine 0.9 mg/dL, serum urea 18 mg/dL, serum potassium 3.5 mEq/L, serum phosphorus 3.3 mg/dL and serum magnesium 0.9 mEq/L (Figure 1).

The patient was followed at the Hematology consultation and completed treatment with another reinduction with cytarabine and daunorubicin, high-dose cytarabine consolidation, and autologous hematopoietic stem cell transplant. She presents chronic cytopenias and hypocellular bone marrow likely due to treatment-related toxicity. However, she is in complete remission from her AML and has no traces of another hematologic neoplasm. After one year of follow-up, laboratory results show normal kidney function and resolution of electrolyte disturbances.

## Discussion

AML is the commonest acute leukemia in the adult population and is caused by the clonal proliferation of a myeloid precursor cell. *NPM1* mutations in the absence of *FLT3-ITD* are usually associated with better outcomes [3].

An association between acute kidney injury and worst renal outcomes, lower complete remission rates, and overall decreased survival in patients with AML undergoing chemotherapy induction has been demonstrated [4,5], emphasizing the importance of early recognition and management of kidney impairment.

Lysozyme-induced nephropathy is a rare presentation of acute tubular necrosis associated with hematologic malignancies [1,6,7]. Lysozyme is a low-molecular-weight (15 kilodaltons) protein produced and stored in monocytes and macrophages. Being part of the innate immune system, lysozyme can lyse cell walls of bacteria and can be detected in human tears, saliva, and nasal secretions, among others [2]. Higher levels of lysozyme are observed in hematologic malignancies with monocytic differentiation, but also in other diseases such as sarcoidosis. Lysozyme migrates in the gamma region by serum protein electrophoresis, producing an increased serum and urinary gamma-globulin concentration in the absence of monoclonal immunoglobulin [7,8]. It is freely filtered in the glomerulus and reabsorbed by the proximal tubules, taken up by endocytosis, and catabolized in phagolysosomes [6,9], and its concentration in the kidney cortex is considerably greater than in the medulla [7]. In the kidney, higher lysozyme concentration has been associated with tubular necrosis, tubular atrophy, degenerative changes, namely cytoplasmatic vacuolization, luminal ectasia, nuclei distortion, loss of brush border, and interstitial fibrosis [2,9]. When the tubular absorptive capability is surpassed, lysozyme urinary concentration increases leading to nephrotic-range nonalbumin proteinuria, also known as lysozymuria [10]. 

Renal manifestations are a reflex of tubular dysfunction - polyuria, hypokalemia, hypophosphatemia, and hypomagnesemia. Some authors postulate that lysozymuria can be associated with Fanconi syndrome and manifestations such as glycosuria and metabolic acidosis may be found [2], but this is still a matter of debate [9]. High serum and urine lysozyme levels are common in patients with monocytic and myelomonocytic leukemias but do not necessarily associate with kidney injury development [11-13].

Despite being a rare and underrecognized cause of acute kidney injury, lysozyme-induced nephropathy explained the constellation of clinical and laboratory findings of our patient. High serum and urinary concentrations of lysozyme further supported this hypothesis. The presence of nonalbumin nephrotic-range proteinuria paralleled by severe hypokalemia, a characteristic finding in lysozyme-induced nephropathy, hypomagnesemia, hypophosphatemia, and polyuria reinforced this diagnosis. Even though a kidney biopsy would establish a definitive diagnosis, it was not performed, because there was a significant hemorrhagic risk and it would not change the treatment choice. In fact, the treatment of lysozyme-induced nephropathy is based on hematologic malignancy treatment [2]. Furthermore, the progressive improvement of kidney function and normalization of electrolyte disturbances during inpatient stay precluded this invasive procedure.

Treatment of AML will improve renal function in lysozyme-induced nephropathy [11]. Treatment aims at complete remission and is based on intensive induction chemotherapy and subsequent intensive postremission therapy. Induction therapy consists of a three-day anthracycline and seven-day cytarabine scheme, frequently known as the “7+3” regimen, and allows complete remission in the majority of young patients. Consolidation therapy consists of high-dose chemotherapy with or without autologous or allogenic hematopoietic cell transplantation. Treatment decisions will depend on factors such as the patient’s age, performance status, and genetic risk stratification [3]. Cytoreduction with hydroxyurea and chemotherapy in association with leukapheresis are useful to reduce tumor burden and prevent leukostasis’ deleterious complications [14]. Since lysozymuria is believed to be a consequence of the increased concentration of monocytes, kidney function, and electrolyte disturbances are expected to improve with leukemia’s cytoreductive therapy [7,15], as was the case of our patient.

This case reinforces the importance of recognizing lysozyme-induced nephropathy as a potential cause of acute kidney injury in acute leukemias. Serum lysozyme is a non-invasive and useful diagnostic tool [7], as it may precociously clarify the etiology of tubular impairment, especially in patients in whom a kidney biopsy imposes an increased and unacceptable risk.

## Conclusions

Renal injury in acute leukemias can involve different pathophysiologic mechanisms and lysozyme-induced nephropathy is an important cause of renal tubular injury. Clinicians need to be conscious of this hypothesis so that an early diagnosis can be made and supportive treatment can be started. Serum lysozyme level is a valuable tool to support this diagnosis and should be considered during the etiological investigation of kidney injury in acute leukemias.
